# *para*-Menthane as a Stable Terpene Derived from Orange By-Products as a Novel Solvent for Green Extraction and Solubilization of Natural Substances

**DOI:** 10.3390/molecules24112170

**Published:** 2019-06-09

**Authors:** Sara Madji, Soukaina Hilali, Anne-Sylvie Fabiano-Tixier, Mathieu Tenon, Antoine Bily, Mickaël Laguerre, Farid Chemat

**Affiliations:** 1 GREEN Extraction Team, Avignon University, INRA, UMR408, F-84000 Avignon, France; sara.madji@alumni.univ-avignon.fr (S.M.); soukaina.hilali@alumni.univ-avignon.fr (S.H.); anne-sylvie.fabiano@univ-avignon.fr (A.-S.F.-T.); 2 Naturex, 250 rue Pierre Bayle, BP81218, F-84911 Avignon CEDEX 9, France; m.tenon@naturex.com (M.T.); a.bily@naturex.com (A.B.)

**Keywords:** *p*-menthane, *Citrus* by-products, hydrogenation, green solvent, extraction, Soxhlet, Dean–Stark

## Abstract

This study aims at investigating *p*-menthane, a novel bio-based solvent resulting from the hydrogenation of d-limonene, as a green alternative to *n*-hexane or toluene for the extraction and solubilization of natural substances. First, conductor-like combination of quantum chemistry (COSMO) coupled with statistical thermodynamics (RS) calculations show a comparable solubilization profile of *p*-menthane and *n*-hexane for carotene, volatile monoterpenes such as carvone and limonene, and model triglycerides. Other data obtained experimentally in solid/liquid extraction conditions further indicate that *p*-menthane showed similar performances to *n*-hexane for extracting carotenes from carrots, aromas from caraway seeds, and oils from rapeseeds, as these products showed a comparable composition. *p*-Menthane was also tested using common analytical extraction procedures such as Soxhlet for determination of oil content via multiple extraction stages, and Dean–Stark for determination of water content via azeotropic distillation. For both systems, yields were comparable, but for Dean–Stark, the distillation curve slope was higher when using *p*-menthane, and the time needed to attain 100% water recovery was 55% shorter than for toluene. Taken together, these results reveal the potential of *p*-menthane as a green replacer for petroleum-based solvents such as *n*-hexane or toluene.

## 1. Introduction

Extraction is a key tool to supply the ever-increasing market requirement for bioactive compounds, and sustains and contributes to the rapid growth of the agro-food industry [1]. Existing extraction technologies are linked to several technological, environmental, and scientific barriers that are generally complex and difficult to overcome, for instance, minimizing energy and toxic solvent consumption, all the while reducing CO_2_ emission and guaranteeing safety and control for both the final product and the technical staff involved [2,3]. Consequently, green engineering and bio-refinery concepts gained a lot of attention worldwide by promoting environmentally friendly and efficient techniques that have the ability to substitute conventional processes [4]. Studies generally include the design of alternative extraction techniques using, for example, microwaves [5], ultrasound [6], or supercritical fluids [7], but may also involve the search for alternative solvents to replace petrochemical solvents [8]. Within this context, it is important to state that solvents are widely used in academia for practical work; in analytical chemistry laboratories; as well as in industries to produce aromas, fats and oils, colors, antioxidants, or fine chemicals. They are regarded as liquid medium used either for chemical product synthesis or for extraction, separation, and purification of bioactive compounds from various plant matrices. Most extraction solvents used in academia or industry, like *n*-hexane, present undeniable advantages including a low boiling point leading to a highly efficient and simple removal, as well as an easy industrial implementation. However, these low boiling point-solvents also generate substantial amounts of volatile organic compounds, explaining why they are universally considered as hazardous with associated environmental and health issues [8,9]. Furthermore, these solvents are often derived from petroleum, a fossil resource. Acknowledging those concerns, many researchers have shifted their focus towards finding green alternatives to substitute petroleum-based solvents [4,10]. Within this context, green analytical chemistry (GAC) emerged to investigate clean practical alternatives to substitute polluting methods in order to lessen the ecological issues resulting from the side effects of conventional analytical methodologies that generate an important amount of chemical waste [11]. One of the solutions is to develop bio-based liquid phases produced from agricultural by-products [4,12,13].

Terpenes, ones of the major secondary metabolite families in plants, have been subjected to multiple investigations as a bio-replacement of conventional solvents [8]. The most common solvent derived from terpenes is undoubtedly d-limonene [4], which is mainly present in *Citrus* peel essential oils, with levels exceeding 70%–95% [13,14,15]. It is responsible for the *Citrus* characteristic scent and could represent a valuable solution to the substitution of petrochemical solvent [14]. Indeed, the world market demand for *Citrus* fruits exceeds 100 million tons. However, up to 40%–50% of the total mass that is treated is considered as peel waste [16,17]. Therefore, valorization of this by-product is of great economic and environmental importance for the industry. Studies that investigated limonene include fats’ and oils’ determination in olive seeds using Soxhlet extraction [18], extraction of oil from rapeseed and microalgae [8,10], carotenoids from tomato [14], aroma from caraway [10], and moisture determination by Dean–Stark distillation [19]. The results obtained from those studies confirmed the potential of d-limonene as a green alternative to replace petroleum solvent in the extraction of secondary and primary metabolites from different plant matrices.

**Figure 1:**
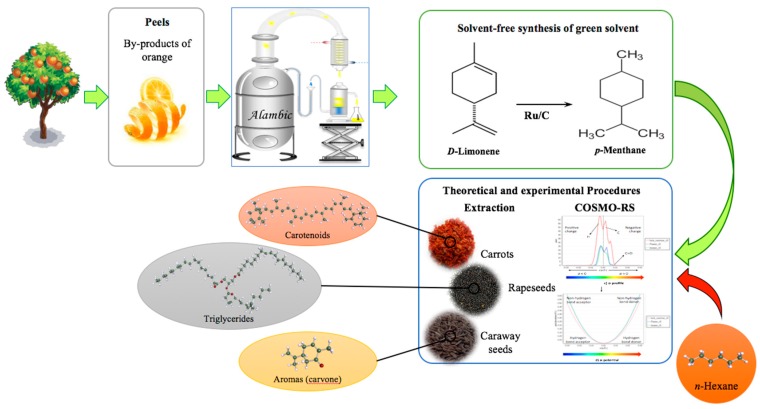
Summary of the followed study protocol. COSMO-RS—conductor-like combination of quantum chemistry coupled with statistical thermodynamics.

While the use of d-limonene as a green solvent may sound like a valuable alternative, the fact that it is an unsaturated terpene that oxidizes easily under exposure to air makes it less attractive, especially as the formed oxidation products are labelled as allergens [4]. Logically, we considered that a total hydrogenation of d-limonene could produce *p*-menthane, a stable saturated terpene, which would not be oxidizable. Such an approach seems particularly relevant if we consider the scarcity of saturated terpenes in natural essential oils compared with the abundance of d-limonene in *Citrus* by-products. This study thus aims to synthesize *p*-menthane (Figure 1) and evaluate its performances under the green analytical chemistry concept as a toluene replacement for moisture determination. The green alternative performance was also compared to that of *n*-hexane for the extraction of natural substances such as carotenoids, aromas, and vegetable oils using Soxhlet or the conventional single step procedure. The idea was to develop an ecological process that takes economical issues into consideration and presents itself as a competitive potential process for natural substance efficient extraction in cosmetic, food, or agrochemical applications. This was completed with a theoretical solubility analysis of targeted secondary and primary metabolites in *p*-menthane and *n*-hexane using a computational predictive method (conductor-like combination of quantum chemistry (COSMO) coupled with statistical thermodynamics (RS)). A comparison of *p*-menthane has also been done with toluene for moisture determination in Dean–Stark distillation apparatus.

## 2. Results and Discussion

### 2.1. Solubility Study: Experimentation and Modelisation

A theoretical study was performed considering the technical parameters of the solvents for the extraction of the targeted compounds via a computational predictive method (COSMO-RS). Solvent selectivity is a matter of polarity-based solubility parameters that involves both solutes and solvents. The software screening model for solvents is a method used to determine and predict thermodynamic properties of the solvent without the need for any experimental data. This is based on conductor-like combination of quantum chemistry (COSMO) coupled with statistical thermodynamics (RS) [20,21].

**Figure 2:**
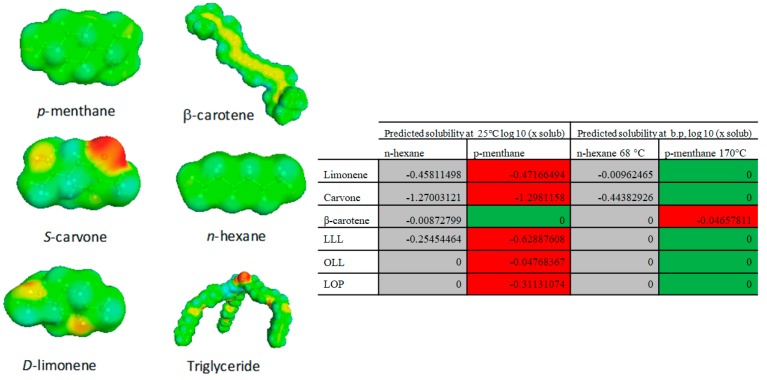
COSMO-RS results of solubility prediction of triglycerides, β-carotene, limonene, and carvone in *n*-hexane 25 °C, *n*-hexane 68 °C, *p*-menthane 25 °C, and *p*-menthane 170 °C. Grey (reference), green (better solvent than reference), red (bad solvent than reference).

Figure 2 shows the COSMO-RS predicted solubilities of (i) β-carotene, (ii) three model triglycerides (LLL, OLL, and LOP; with L, O, and P for linoleoyl, oleoyl, and palmitoyl chains, respectively), and (iii) d-limonene and S-carvone aroma compounds in *n*-hexane and *p*-menthane. These targeted solutes were chosen as they represent valuable compounds extractable from carrots, rapeseeds, and caraway, respectively. The simulations were carried out by taking into consideration the solvent boiling points (b.p.) and the operating laboratory conditions set at 25 °C. The results expressed in log10 (x-soluble) show a high theoretical solubility of the compounds in both solvents. In most cases, *p*-menthane has probability of solubility higher than, or similar to, *n*-hexane, which represents a significant first step to determine whether *p*-menthane can successfully replace *n*-hexane for the extraction of different natural products.

“x-soluble”: solute/solvent ratio expressed as a mole fraction. Gray: reference. Green: better or equivalent. Red: slightly poorer than the reference. Triglycerides abbreviated use L, O, and P for linoleoyl, oleoyl, and palmitoyl.

### 2.2. Extraction of Aromas from Caraway Seeds

Food aromas are complex mixtures of volatile substances. They are usually only present at very low concentrations. Before using aromas, an extraction process from the targeted matrix is required.

A study conducted by Filly et al. [22] compared the performance of eight alternative solvents (α-pinene, MeTHF, ethyl acetate, ethyl lactate, ethanol, dimethylcarbonate, and water) to n-hexane for extraction of aromas from caraway seeds. The results showed that bio and green solvents have the potential to replace petroleum-based solvents. Here, *p*-Menthane was evaluated as an alternative to *n*-hexane for the extraction of aromas from caraway seeds using solid–liquid extraction by ULTRA-TURRAX^®^ (Tube Drive P control for stirring, dispersing, grinding, and homogenization).

The chemical selectivity and performance comparison between the two solvents for two major compounds that are d-limonene and S-carvone were determined using GC-MS (gas chromatography coupled to mass spectrometry). Several authors established that those two monoterpenes are the major compounds of caraway seeds in extractions carried out with different solvents [4,23]. As illustrated in Table 1, it is interesting to notice that both solvents showed a relatively similar profile of aroma compounds with no particular solvent selectivity detected. The major compounds extracted from caraway seeds were S-carvone with approximately 60% for *p*-menthane and 64% for *n*-hexane, followed by d-limonene with 40% and 35% for *p*-menthane and *n*-hexane, respectively.

### 2.3. Carotenoids Extraction from Carrots

Carotenoids are largely used in the food industry as colorants or nutritional ingredients [24]. Several studies have indeed shown the beneficial role of carotenoids in decreasing the risk of diseases [25] such as heart diseases, cardiovascular diseases, and cataracts [22,26,27]. Carrot is considered as an important dietary source of carotenoids, mainly carotenes, which impart their characteristic orange color to carrots. As carotenes are one of the most hydrophobic compounds in the plant kingdom (logP around 15), they cannot be extracted with aqueous solutions of whatever organic solvent. Existing methods of carotene extraction are thus mostly based on pure organic solvents such as *n*-hexane.

Green extraction techniques were developed according to the literature for carrots carotenoids extraction, such as ultrasound irradiation [28] and supercritical CO_2_ extraction with ethanol [29] or canola oil [30] as co-solvent for the recovery of carotenoids. The high achieved extraction yield for both processes may suggest the positive feasibility of the processes.

Here, the objective was to investigate *p*-menthane as a bio-sourced alternative via solid–liquid extraction using an ULTRA-TURRAX^®^ device. Extractions were performed in the same maceration conditions for both solvents for one hour at room temperature.

Table 1 shows that a slightly higher yield was obtained for the extraction of α- and β-carotene using *p*-menthane compared with *n*-hexane, although the differences were modest. These results are somewhat in agreement with the COSMO-RS predicted solubilization properties of both solvents for β-carotene (Figure 2). Therefore, for this specific application, *p*-menthane could be considered as a promising alternative to *n*-hexane.

### 2.4. Rapeseed Oil Extraction

Rapeseed oil is an internationally consumed commodity oil, ranked third worldwide and first in Europe [4]. Generally, the industrial processing of rapeseed involves pressing followed by extraction with hexane. In fact, pressing as a single unit operation is relatively inefficient and does not ensure complete expulsion of oil. Therefore, solvent extraction is an additional operation for an efficient extraction [31]. The outcome is two products: oil and low-valued meal. This latter is either used for animal feed or fertilizer. However, this method presents numerous industrial disadvantages as using hexane for rapeseed oil extraction raises many safety and environmental concerns as hexane is listed as a hazardous air pollutant [32] and is known as a potential neurotoxic. Considering those concerns, oil extraction of rapeseeds was carried out by solid–liquid extraction using ULTRA-TURRAX^®^ for *p*-menthane and *n*-hexane.

Table 1 shows the composition of rapeseeds oil determined by GC-FID (gas chromatography techniques coupled to flame ionization detector) and extracted using both solvents. The lipid profiles of *p*-menthane and *n*-hexane extracts were similar in terms of fatty acid composition. The percentage summations of saturated, as well as mono- and poly-unsaturated fatty acids, and yields were also compatible with those of rapeseed oils documented in literature for *n*-hexane, methyl tetrahydrofuran, ethyl acetate, *p*-cymene, *d*-limonene, and pinenes [4,33].

### 2.5. Analytical Extraction: Soxhlet Extraction of Rapeseeds

Soxhlet is globally the most used lab-scale technique for fat and oil extraction. However, it is time- and energy-consuming and also uses important amounts of petroleum-based solvents, mainly *n*-hexane [18]. As mentioned above, the latter is ranked high on the hazardous solvents list. Consequently, many research groups over the years have investigated new alternatives [4]. In this context, bio-solvents such as terpenes have gained a great deal of attention as they can potentially offer a renewable alternative to these solvents [34].

**Figure 3:**
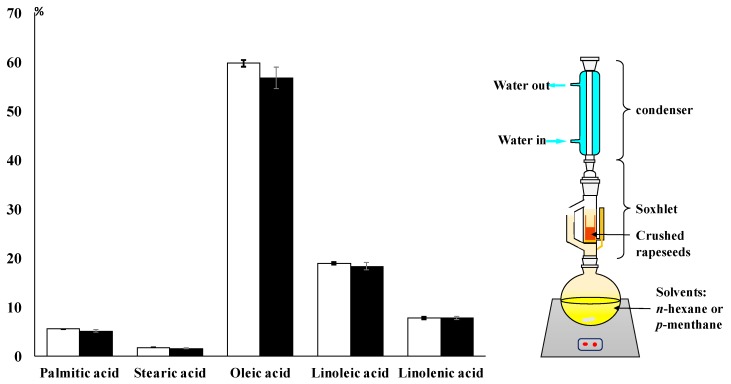
Fatty acid composition of rapeseed oils extracted with *p*-menthane (◼) and *n*-hexane (◻).

The extraction results on rapeseeds are shown in Figure 3. The mass yield of *p*-menthane extraction was compared with that of *n*-hexane (40.5% vs. 39.5%, respectively). No significant difference was noted, which may be the result of the strong polar nature of both solvents that leads to a high dissolving power for triglycerides. Additionally, *p*-menthane has a higher boiling point, inducing a decrease of oil viscosity and giving a better diffusion rate in the rapeseed matrix.

Fatty acid methyl ester’s (FAME’s) derivatives were identified and quantified by GC-FID. Values of ∑SFAs (total saturated fatty acids) were 14.1% for *p*-menthane and 14.9% for *n*-hexane. Amounts of ∑MUFAs (total mono-unsaturated fatty acids) were 61.1% for *p*-menthane and 58.1% for *n*-hexane, while those for ∑PUFAs (poly-unsaturated fatty acids) were 26.6% for *p*-menthane and 26.0% for *n*-hexane.

These results point out that for both solvents, the fatty acid profile of the resulting oils (the composition) and the mass yields of the extraction process are very similar. The main fatty acids extracted using both solvents are oleic (C18:1), linoleic (C18:2), and linolenic (C18:3) acids. These three unsaturated fatty acids represent more than 85% of the total extracted oil fatty acid composition. Even though *n*-hexane is widely used for vegetable oil extraction using Soxhletin analytical laboratories because of its high extraction and selectivity abilities, *p*-menthane exhibited similar capacities, making it a competitive and efficient alternative.

### 2.6. Analytical Extraction: Dean–Stark Azeotropic Distillation of Carrots

Initially, Dean–Stark apparatus was developed to measure the water content present in petroleum products, which is feasible thanks to the azeotropic ability of numerous compounds [35]. Nowadays, Dean–Stark apparatus has become a tool associated with the separation of water from different matrices. The latter has repeatedly been modified to cover a broader range of applications. It is widely used for water determination in food products and is frequently cited for products with volatile compounds. The azeotropic distillation occurs between water and petroleum solvents such as toluene [4]. While toluene gives satisfying results, it is labeled as hazardous and toxic [19,36]. To mitigate environmental and health concerns related to this solvent, new alternative were considered [37,38]. In this way, a range of terpenes were investigated including d-limonene [19], and the results showed that there are no significant differences between toluene and d-limonene procedures, thus concluding that d-limonene could be considered as a possible alternative for petroleum-based solvents.

**Figure 4:**
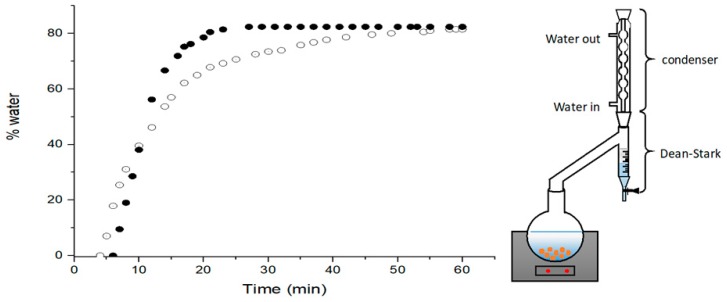
Water distillation kinetics of fresh carrots via toluene (⚪) and *p*-menthane (⚫) using Dean–Stark.

Figure 4 shows water distillation kinetics of carrots for both toluene and *p*-menthane. The azeotropic distillation of the mixture has different kinetics for water recovery for each solvent because of the boiling point difference. Water distillation kinetics were somewhat similar for the two solvents. When using *p*-menthane, the water recovery was delayed by 2 min. However, once the distillation started, the alternative solvent gave a faster water recovery compared with toluene. The boiling point of the azeotropic mix is very close to the water boiling point; the water is accordingly more volatile as to lower temperatures. The distillation curve slope was higher when using *p*-menthane, whereas the time needed to attain 100% water recovery was 55% shorter than for toluene. Thus, *p*-menthane will reduce not only the procedure duration, but also the energy consumption. The percentage of carrot water content obtained through this process was 81.5% and 82.4% for toluene and *p*-menthane, respectively, which means that *p*-menthane could possibly replace toluene in a Dean–Stark procedure.

## 3. Materials and Methods

### 3.1. Plant Material and Chemicals

Rapeseeds were provided by OLEAD. They originated from France-Gironde and were cultivated in 2016. Caraway seeds and carrots were purchased from a local market (Avignon, France). For chemicals, *n*-hexane (HPLC grade) was supplied by VWR International (Darmstadt, Germany). Standards such as β-Carotene (95%–99%) and d-limonene were provided by Sigma-Aldrich (Germany), and S-carvone was from Merck™ (Darmstadt, Germany). Ruthenium on carbon (5 wt%) was purchased from Strem Chemicals.

### 3.2. Hydrogenation of d-Limonene

Hydrogenation of d-limonene to *p*-menthane at a low temperature (approximately 40–50 °C) was carried out using a ruthenium catalyst on activated charcoal, thus leaving a great margin to control the exothermicity of the reaction (boiling points of d-limonene and *p*-menthane are 177 and 170 °C, respectively). A catalytic charge of 1.5 wt% ruthenium on charcoal (5 wt%) was sufficient to completely hydrogenate limonene under 25 bars of hydrogen pressure. Those tests were conducted on a maximum batch of 56 g of *d*-limonene. All reactions were conducted until complete conversion of d-limonene to *p*-menthane was achieved, the purity of which was determined by gas chromatography. The products were all mixed and then filtered under vacuum on Clarcel (filter aid) to retain the catalyst and give a completely colorless liquid product. The overall mass yield of the operation was 97%. Losses could be attributed to transfers and the amount of material retained on the filter during the removal of the catalyst.

### 3.3. Computational Solubility Assessment

The solubility of (i) *β*-carotene from carrots, (ii) S-carvone and d-limonene aromas from caraway seeds, and (iii) model triglycerides (glyceryltrilinolein, LLL; glyceryloleoyl–dilinoleoyl, OLL; and glyceryllinoleoyl–oleoyl–palmitoyl, LOP) from rapeseeds was evaluated for both *p*-menthane and *n*-hexane using the theoretical prediction model COSMO-RS (COSMO software). Computation methods consider the technical properties of the solvents to assay and examine the solutes. The chemical structures of the solvents and solutes were transformed by JChemPaint version 3.3 (GitHub Pages, San Francisco, CA, USA), while for the entry syntax, (SMILES) notation was followed. They were then used for the solubility parameters calculation of solvents and targeted compounds. Predictions of relative solubility were carried out at two temperatures: 25 °C and at the boiling point of each solvent (extraction temperature).

### 3.4. Solid–Liquid Extraction

Extractions of the oil from rapeseed, carotenoids from carrots, and aromas from caraway seeds were performed using an ULTRA-TURRAX^®^ Tube Drive System (Paris, France) [4,39]. The dried vegetable materials were grounded prior to extraction. One gram of each matrix, 20 g of ceramic balls, and 10 mL of solvent (*p*-menthane or *n*-hexane) were added into a tube with a stirring device. The obtained mixture was mechanically stirred and macerated at 4000 rpm for 60 min at 25 °C. The residue was removed by filtration and centrifuged at 9000 rpm for 10 min.

### 3.5. Dean–Stark Distillation

At first, moisture determination of carrots was carried out according to the American Oil Chemists’ Society (AOCS) official method Ja 2a–46 [40]. The experimental apparatus was composed of three major parts: a 500 mL round-bottom flask coupled with an electrical heater, a graduated Dean–Stark receiver, and a condenser. The sample weight was 10 ± 0.6 g for each experiment. The weight amount was set according to Harel and Talmi [41] to obtain an adequate water amount in the receiver that did not exceed 10 mL, which was the maximal countenance. Toluene as a distillation solvent was compared to *p*-menthane. One hundred milliliters of each were used.

The electrical heating was maximized in the beginning of the experiment until the first droplets in the Dean–Stark receiver were collected. The heating was then adapted to a distillation rate of 100 drops/min. When most of the water was distilled (approximately 80%), heating was then increased to a distillation rate of 200 drops/min and continued until the end of the distillation. About 30 mL of either toluene or *p*-menthane was added at the top of the condenser to recover the water droplets that adhered to the condenser. The Dean–Stark collector was then cooled to room temperature for a few minutes until the layer was clear.

The moisture content was calculated as follows:

(1) $$Moisture\;\left(\%\right)=\frac{\mathrm{volume}\;\mathrm{of}\;\mathrm{water}\;\left(\mathrm{mL}\right)\times 0.997\times 100}{\mathrm{Weight}\;\mathrm{of}\;\mathrm{fresh}\;\mathrm{sample}\;\left(g\right)},$$

where 0.997 is the water density at 20°C.

### 3.6. Soxhlet Extraction and Procedure

The extraction of oil from rapeseeds was carried out according to the standardized Soxhlet procedure [18] using *n*-hexane and *p*-menthane as solvents. For each extraction, 20 g of dried and ground rapeseeds was put in a 33 × 100 cellulose thimble and inserted into a 250 mL Soxhlet apparatus. Experiments were carried out for eight hours with a solvent volume of 170 mL. After the extraction, the solvent was eliminated in a rotary evaporator. The content was then dried for 2 h in an oven under a circulating air set at 65 °C. After cooling in a desiccator for 1 h, the flask was precisely weighed up to constant value. The results obtained were expressed as follows:

(2) $$\%\;Oil\;content=\frac{\mathrm{weight}\;\mathrm{of}\;\mathrm{oil}\;\mathrm{obtained}\;\mathrm{after}\;\mathrm{extraction}}{\mathrm{weight}\;\mathrm{of}\;\mathrm{dry}\;\mathrm{sample}}\times 100.$$

### 3.7. Total Carotenoid Analysis

Total carotenoids were quantified using UV/visible spectrophotometry (Biochrom Libra S22 UV/Vis spectrometer, Guibeville, France). Absorbances were measured at 450 nm using *n*-hexane as a blank. Carotene concentration in each extract was calculated via a calibration curve previously obtained using a pure standard of β-carotene (>98%). The carotene yield was expressed as g (β-carotene) per 100 g of dry matter (%).

### 3.8. Specific Determination of Carotenoids by HPLC-DAD

The identification and quantification of extracts was carried out using HPLC-DAD consisting of a HPLC Agilent 1200 equipped with a UV/vis detector (DAD). Separation was attained at a temperature of 25 °C on a YMC C30 column (250 × 4.6 mm and 3 µm ID). The mobile phase in this case consisted of a combination of A (methanol/MTBE/water, 81/15/4) and B (methanol/MTBE/water, 10/90/4). The linear gradient was from 100% to 0% B (*v/v*) within 90 min. The pumping flow rate was 1.0 mL/min. The volume used for the sample volume was 5 µL, while the quantitative detection was measured at 450 nm. To establish carotenoids calibration curves, standard carotenoids were used with concentrations ranging from 5, 10, 25, 50, to 100 mg/L. Identification of the main carotenoids in carrot extracts was carried out in triplicate by comparing the retention times and absorption spectra.

### 3.9. Rapeseed Oil Profiling by GC-FID Analysis

Rapeseed oil FAMEs were separated, identified, and quantified by GC-FID. Preparation of samples from extracted rapeseeds oils was performed using acid-catalyzed trans-methylation [4]. Then, 1 mL of methanolic sulfuric acid (5% *v/v*) was added to a precise volume of extracted oils and the internal standard. The mixture was then heated at 85 °C for 90 min and latter onset to cool until it reached room temperature. Then, 1.5 mL sodium chloride (0.9%) solution and 1 mL *n*-hexane were added. The mixture was shaken strongly for 30 s prior to centrifugation at 4000 rpm for 2 min. An aliquot of the organic layer was moved into a vial for a GC analysis using a 7820A GC system (Agilent technologies, Santa Clara, CA, USA) equipped with a BD-EN14103 capillary column (30 m × 0.32 mm × 0.25 μm), as well as an FID and an auto-sampler. The carrier gas was helium at 33 mL/s. The sample injection volume was 2 μL, while the split ratio was set at 1:20 at 250 °C. The oven initial temperature was set at 50 °C for 1 min, and then increased to 20 °C/min until it reached 180 °C, then a rate of 2 °C/ min until 230 °C, and was then held isothermally at 230 °C for 10 min. Data collection was carried out by Agilent EZChrom Elite software. Identification of FAMEs was performed in comparison with purified FAME standards (Sigma Co., St Louis, MO, USA), while the quantification was achieved using internal calibration. The internal standard used was glyceryl triheptadecanoate (Sigma Co., Cleveland, OH, USA).

### 3.10. Identification of Aroma by GC-MS

Aroma identification was carried out using an Agilent 6890N GC coupled with an Agilent 5973 MS (Agilent, Massy, France). The extracts were analyzed on a fused-silica capillary column HP-1MSTM (polydimethylsiloxane, 50 m × 0.25 mm i.d. × 0.25 µm film thickness; Interchim, Montluçon, France) and INNOWAX (polyethyleneglycol, 50 m × 0.20 mm i.d. × 0.4 µm film thickness; Interchim, Montluçon, France). The carrier gas was helium pumped at a constant flow of 1 mL/min. The injector temperature was set at 250 °C with split ratio of 1:150. The initial temperature was set at 45 °C and increased by a rate of 2 °C/min until 250 °C, and was then held isothermal for 20 min at either 250 °C (apolar column) or 230 °C (polar column). The temperature of 230 °C is the ion source temperature (apolar column) and 250 °C corresponds to the transfer line temperature (polar column). The ionization energy was set at 70 eV.

## 4. Conclusions

In this work, the potential of *p*-menthane as a green alternative to petroleum solvents such as *n*-hexane and toluene was investigated. This bio-based solvent was prepared by hydrogenation of D- limonene, a by-product of deterpenation of essential oils obtained from orange peels. The solubility of certain bioactive compounds in *p*-menthane was experimentally and theoretically compared with *n*-hexane using a simulation software COSMO-RS. It was found that both solvents have relatively similar solvation efficacy. Experimentally, it was observed that a slightly higher extraction yield can be obtained on carrots using the alternative solvent, while for caraway aromas and rapeseed oils, similar extraction potential was recorded for both solvents. *p*-Menthane was also used for water determination using Dean–Stark apparatus. As the boiling point of *p*-menthane is higher than that of toluene, the azeotropic distillation is consequently higher for *p*-menthane, leading to a brief delay of the start of the distillation; but once the distillation started, the recovery was 55% shorter than for the toluene. Thus, *p*-menthane could offer an interesting green and sustainable alternative solution to replace some hazardous petroleum-based solvents for a diverse set of applications ranging from practical work for students in academia, to analytical laboratories and industrial processes in the pharmaceutical, cosmetic, aroma, and food areas.

## Figures and Tables

**Table 1:** Comparison of efficiency using *n*-hexane and *p*-methane for extraction of secondary and primary metabolites extracted from different plant matrices. GC-MS—gas chromatography coupled to mass spectrometry; DM—dry matter; FID—flame ionization detector; SFAs—saturated fatty acids; MUFAs—mono-unsaturated fatty acids; PUFAs—poly-unsaturated fatty acids.

Description	*n*-Hexane	*p*-Menthane
**Caraway Seeds**	**Major Compounds Identified by GC-MS (µg/mL)**	
d-Limonene	622.3 ± 49	624.6 ± 32
*S*-Carvone	1118 ± 33	934 ± 29
Yield (g/100 g)	1.74 ± 0.08	1.56 ± 0.03
**Carrots**	**Carotenoids Identified by HPLC (µg/mL)**	
β-Carotene	28.17 ± 0.23	25.97 ± 1.96
α-Carotene	11.01 ± 1.51	11.24 ± 0.23
Yield (g/100 g DM)	32.5 ± 2	35.7 ± 1.5
**Rapeseed Oil**	**Compounds Identified by GC-FID (%)**	
saturated compounds		
Myristic acid C14:0	0.09 ± 0.0004	0.09 ± 0.0002
Palmitic acid C16:0	5 ± 0.038	4.9 ± 0.063
Stearic acidC18:0	1.5 ± 0.011	1.5 ± 0.018
Arachidic acid C20:0	0.6 ± 0.003	0.6 ± 0.006
Behenic acid C22:0	0.4 ± 0.002	0.4 ± 0.003
Lignoceric acid C24:0	0.2 ± 0.001	0.2 ± 0.002
mono-unsaturated		
Palmitoleic acid C16:1	0.2 ± 0.001	0.2 ± 0.003
Oleic acid C18:1	62 ± 0.43	62.1 ± 0.8
Gadoleic acid C20:1	1.2 ± 0.007	1.2 ± 0.01
poly-unsaturated		
Linoleic acid C18:2n6c	19.5 ± 0.14	19.4 ± 0.25
Linolenic acid C18:3n3	8.9 ± 0.06	8.8 ± 0.11
∑SFAs	7.9	8
∑MUFAs	63.5	63.6
∑PUFAs	28.5	28.3
Yield (g/100 g)	38.38 ± 0.05	37.1 ± 0.07
